# Key Insights into Developing Qualitative Concept Elicitation Work for Outcome Measures with Children and Young People

**DOI:** 10.1007/s40271-023-00663-6

**Published:** 2024-02-14

**Authors:** Samantha Husbands, Paul Mark Mitchell, Joanna Coast

**Affiliations:** Health Economics Bristol, Population Health Sciences, Bristol Medical School, University of Bristol, Bristol, BS8 1NU UK

## Abstract

Qualitative concept elicitation can develop meaningful patient-reported outcome measures for children and young people; however, the methods used for concept elicitation are often underreported for this population. This paper provides in-depth insight into the methods used for concept elicitation with children and young people, with a focus on key stages of concept elicitation that are challenging or unique to doing this research with children. Drawing on our experiences of developing wellbeing measures for children and young people aged 6–15 years, we detail the processes followed in our qualitative concept elicitation work, covering issues related to sampling and recruitment, encouraging informed assent and freedom over children and young people’s involvement in concept elicitation, and the use of creative and participatory methods to develop measure items. We provide reflections on the approaches taken to navigate challenging aspects of concept elicitation with children and young people. Our reflections suggest that using existing links and online recruitment methods can help to navigate organisational gatekeepers, and using appropriate processes to develop study information and obtain informed assent can ensure that research is inclusive and that children have the freedom to decide whether to be involved. Our adaptation of a creative and participatory activity to generate concepts for measure items suggests that such approaches can be engaging and may help to give children greater control over their participation. In detailing our methods, we hope to have developed a useful resource for other researchers, while highlighting the value of transparent reporting in this area.

## Key Points for Decision Makers

**Table:** 

This paper focuses on providing detailed insights into key stages of concept elicitation with children and young people, particularly those that are challenging or unique to doing concept elicitation with children.
Sampling and recruitment, encouraging informed assent and children and young people’s freedom over their involvement, and using creative and participatory methods to develop measure items are aspects of concept elicitation with children and young people that have previously been underreported.
Reflections on our own approaches to concept elicitation with children and young people include ways to navigate gatekeepers during recruitment, processes to encourage inclusive and informed participation in research, and the adaptation of a creative and participatory activity to engage children and to facilitate high-quality data collection for item generation.
Concept elicitation work with children and young people would benefit from greater transparency in reporting to allow researchers to learn from each other’s processes.

## Introduction

In healthcare decision making, choices must be made about which health and social care interventions to fund from limited resources [1]. National decisions in many countries are taken with the aid of economic evaluation, which compares the clinical and cost effectiveness of competing interventions. Patient-reported outcome measures (PROMs) are often used to collect outcome data that feed into these evaluations. PROMs are questionnaires designed to collect information on outcomes from the perspectives of patients and other target populations themselves. These are completed pre- and post-intervention to evaluate interventions and services [2]. PROMs can be disease-specific or generic, with the latter commonly being used in health and social care decision making to compare interventions across a broad range of contexts [3]. Literature on PROM development emphasises the need for measures to be relevant and responsive to changes in the outcomes of the population of interest, including that it has content validity [3]. Guidance suggests that the use of direct, qualitative research with the target population to develop measure items (referred to herein as concept elicitation) is key to establishing measures that contain valid and meaningful content [4]. Furthermore, reporting concept elicitation processes in full is important to providing evidence of this content validity, by demonstrating the connection between the concept that is being measured and the population of interests’ understandings and lived experiences of that concept [4].

The importance of using qualitative research for concept elicitation is similarly emphasised in the development of PROMs for children and young people (CYP) [5, 6]. However, direct research with CYP for PROMs development is challenging, including issues with CYP’s understanding and perceived ability to engage in concept elicitation exercises [6–8]. Other challenges include recruiting CYP via gatekeepers [9, 10] and ensuring ethically appropriate research processes between children and adult researchers. In a recent systematic review, issues were raised with the quality of reporting of concept elicitation with CYP; low-quality methods reporting meant that it was difficult to make judgements around the validity of concept elicitation [8]. This finding was supported by a similar scoping review [11] and another study suggesting that limited transparency in reporting qualitative methods for CYP measure development prevented in-depth understanding of how to optimally undertake concept elicitation with children [12]. Such studies suggest that enhanced reporting of robust processes for doing concept elicitation with CYP would be beneficial [8, 11, 12].

This paper aims to provide detailed insight into key stages of concept elicitation with CYP for PROM development, focusing on issues that are particularly challenging and/or unique to doing this research with CYP. These key stages are also those that lacked detailed reporting in previous studies, including sampling and recruitment of CYP, data collection using creative and participatory (CAP) methods and ethics and reflexivity during the research process [8, 11]. The insights are based on lessons learnt through our own research to develop wellbeing measures for use with CYP in economic evaluation [13, 14]. We hope that being transparent about how we have undertaken aspects of concept elicitation with CYP will be helpful to other researchers, while also emphasising the value of transparency in reporting concept elicitation.

## Process of Concept Elicitation

### Measure Development Background

The concept elicitation work here is part of a national study aimed at generating generic capability wellbeing measures to be used across childhood for CYP aged 0–18 years [13]. The measures focus on CYP’s outcomes in terms of capability wellbeing, namely what a person can do or be in their life [15]. This paper concentrates on the process of concept elicitation for measures for CYP aged 6–15 years and covers some of the qualitative development of the measure items but does not detail methods for cognitive interviewing for content validity, which are explored elsewhere [6, 16]. We focus on the process of undertaking qualitative in-depth interviews with CYP to develop the concepts to inform the items in our measures. The following sections of the manuscript present aspects of concept elicitation with CYP that typically encompass additional practical challenges when compared with concept elicitation with adults. We provide in-depth descriptions of the methods and approaches that we used in our own work to navigate these challenges. The stages we discuss next are sampling and recruitment of CYP, encouraging informed assent and CYP’s freedom over their involvement in concept elicitation, and the use of CAP methods to facilitate high-quality data collection.

### Sampling and Recruitment of Children and Young People (CYP)

Sampling and recruitment are key to ensuring content validity of concept elicitation, given the requirement for measure items to be developed with, and meaningful to, the populations in which they will be used [4, 5]. This is particularly important for CYP measures, where there are likely differences between what is important to specific age groups of CYP, potentially requiring the development of separate measures with different measure items [5, 7]. Furthermore, a previous study reported that measures were being recommended for age groups that had not been included in concept elicitation work [8]. In our development of the capability measures, we therefore ensured that we had representation for CYP from all age groups in our target population, to allow our analysis of the empirical data to indicate whether differences in the responses of CYP of different age groups could indicate a need for multiple measures. Our measures are intended for use across the whole population within each age group and thus we aimed to sample a broad range of CYP to represent the characteristics of those who will complete each measure. We also considered which CYP characteristics could potentially lead to differences in what was considered important to wellbeing, thus potentially generating different measure items. We took a purposeful, maximum variation approach to sampling [17] and considered the widest range of perspectives and experiences relevant to wellbeing that might exist within the general CYP population. We sampled CYP of different ages, levels of deprivation, sex, ethnicity, household composition and health status [13].

After developing a sampling strategy, we found recruiting CYP to be challenging, specifically navigating gatekeepers. Such gatekeepers can typically include professional organisations, professional gatekeepers and also parents/guardians [10]. Although gatekeepers undoubtedly protect the interests of CYP, they can be a barrier to recruitment, making it important to factor in time in the study for this. We found that recruitment was speedier and more successful where we had existing links with gatekeepers; for example, where our university had established relationships with local schools. However, we did also develop connections by contacting organisations without existing links directly and thus found it beneficial to try multiple avenues of recruitment. When approaching gatekeepers, we found it helped to be clear about what would be required of them in terms of recruitment and to be flexible to fit with their existing commitments.

The emergence of coronavirus disease 2019 (COVID-19) in 2020 required us to move recruitment online due to the closure of recruiting organisations. Despite the upheaval, this proved to be beneficial to recruitment. We recruited parents/guardians using Facebook and its ‘targeted ads’ feature, which allowed us to promote our recruitment advert specifically to users who had children. The benefits of recruiting online included sampling CYP directly through parents/guardians, avoiding professional gatekeepers, and recruiting a broader range of CYP relatively quickly compared with recruiting via local organisations, including those in different geographical locations [18].

### Encouraging Informed Assent and CYP’s Freedom Over Their Involvement in Concept Elicitation

Considerations for undertaking concept elicitation with CYP include those related to developing ethically appropriate research processes. This includes ensuring that CYP are confident enough to engage with and share their voices during research; for example, by minimising power differentials between CYP and adult researchers [10, 19], and gaining appropriate informed consent/assent from CYP. More information on our use of CAP methods to address power imbalances in our concept elicitation work is given in Sect. 2.4.

To encourage informed assent and CYP’s freedom over their involvement in the study, we took time to develop CYP information sheets, with the format and presentation of information specifically designed to facilitate CYP’s understanding of what research participation would entail [21, 25, 26]. Images and simple and clear language were used, and different versions of information sheets were originally created for different age groups of CYP (a total of four information sheets to cover the age range 6–15 years). These information sheets were piloted with a young person’s advisory group (YPAG), with CYP aged 10–17 years. YPAG members were asked to read drafts of the information sheets and feedback in a 1-h meeting with the research team. Suggestions focused on making the information sheets more comprehensive and accessible to all CYP. Key feedback from the YPAG included labelling information sheets according to the reading ages appropriate to school key stages in the UK rather than by age group, so as not to exclude any CYP who may have a lower reading level to their chronological age. The YPAG generally had positive comments on the presentation of the information sheets but made minor suggestions to improve the wording to make them more inclusive, including changing mentions of parents to also include ‘guardian’, ‘carer’ and ‘the people you live with’. All suggested changes were made in line with YPAG feedback.

We also developed written assent forms to allow CYP to give permission for their own study involvement (in addition to informed consent from parents/guardians) and to allow CYP additional opportunity to consider and reflect on their participation [21]. Prior to any data collection, CYP were provided with an appropriate study information sheet and asked to complete an assent form to indicate that they were happy to take part in the study. The assent form contained five questions and CYP were required to tick ‘yes’ or ‘no’ for each question. The questions were ‘Do you understand what the project is about?’; ‘Have you asked all the questions you want?’; ‘Have you had your questions answered?’; ‘Do you understand you can stop the study at any time?’; and ‘Are you happy to take part?’. Depending on whether interviews were carried out online or in person, all CYP were given time to read the information sheet and complete the assent form, either receiving the documents in advance of the interview or being given dedicated time before an interview to read the study information. The study information sheets for CYP aged under 16 years suggested that a parent, guardian, or carer read it through with them, but time was also taken by the researcher prior to each interview to explain the study to each CYP, including what their involvement would entail and providing opportunity for questions. If a child or young person answered ‘no’ to any of the questions on the assent form, they were asked not to sign their name on the form and instead talk any concerns or questions that they had through with the researcher before deciding whether to participate. It was considered important that CYP were given as long as they needed prior to beginning an interview to decide whether they wanted to take part. Generally, gaining assent from CYP took around 5 min, as the majority of CYP informants did not have any questions or concerns for the researcher when asked and had already reviewed the study information with their parent prior to the interview. Many of our CYP sample had also received the assent form in advance of the interview and had completed this beforehand. It is worth noting that the time taken to gain assent may eat into the time that CYP are able or willing to engage in the research itself, particularly when undertaking research with younger CYP [7], and thus it is important to account for this additional time when planning research processes. Reflecting on our own processes, we found that sending study information and assent forms in advance of data collection appeared to facilitate CYP’s comfort with, and understanding of, the study and what was being asked of them, and thus likely contributed to less time being needed to complete the assent procedures. All CYP were reminded before the interview that they could stop taking part at any time without having to provide a reason.

A final consideration unique to doing concept elicitation with CYP is the role of parents/guardians in interviews, with guidelines suggesting that parent involvement in CYP’s data collection should be minimised to avoid influencing CYP’s responses [6, 7]. To ensure that both parents/guardians and CYP were comfortable with study participation, we allowed decisions regarding parents’ presence during interviews to be made by the parent and CYP together. However, parents were asked by the researcher not to influence the CYP during data collection or speak on their behalf. In our experience, most parents and CYP were happy for the child to be interviewed independently, although it was more difficult to regulate parental involvement when interviews moved from in-person to online, making it important to be transparent about their potential influence over data collected.

### The Use of Creative and Participatory Methods to Facilitate High-Quality Data Collection

Reflexivity requires researchers to reflect on how their involvement in, and decisions during, studies can influence research processes and findings [20]. In concept elicitation with CYP, this involves considering how relationships between adult researchers and CYP might influence final measure items. A key issue is ensuring that CYP’s voices are maintained during the research to feed directly into measure items [21], to lessen the potential for power imbalances or researcher assumptions biasing the content of the measure. In our study, balanced power relations were encouraged through the development and use of a CAP activity, which facilitated CYP having a sense of control over the direction of the research [22, 23], as it allowed them to talk through their finished maps during data collection rather than only answering researcher questions. Allowing CYP to lead data collection facilitated open-ended questioning for the child’s narrative of what was important [24] and minimised researcher assumptions about what the child had drawn or written.

Choosing an appropriate data collection method for concept elicitation involves selecting an approach to allow in-depth understanding of the concepts important to a population in terms of the outcome(s) of interest [6, 27]. For CYP, it is important to ensure that the method is engaging and can generate rich data to inform valid measure items. Existing guidance for CYP PROMs compares interviews with focus groups for concept elicitation, noting the impact of each method on CYP engagement [6, 7]. However, these resources offer less exploration of the use of CAP methods to enhance CYP engagement in concept elicitation, and opinion on their use appears divided. For example, the ISPOR task force guidance for paediatric PROMS [6] suggests that drawing could be potentially useful in concept elicitation, particularly to engage younger children, but other guidance suggests that such methods should be used cautiously because they can distract from the purpose of the research [7].

In our development of the capability measures, we decided to use a creative activity with CYP alongside concept elicitation interviews. Compelling reasons for using CAP methods include engaging CYP who might have problems with communicating using only language-based methods such as interviews and focus groups [10, 23, 28] and giving CYP the time, space and freedom to immerse themselves in research and provide in-depth and considered responses [23, 29]. Several CAP methods are available, including drawings, photographs, diary keeping, guided tours, and mapping and activity packs [9, 28, 30–32]. In our study, we selected elements from several of these methods, alongside hierarchical mapping techniques [33, 34].

**Fig. 1:**
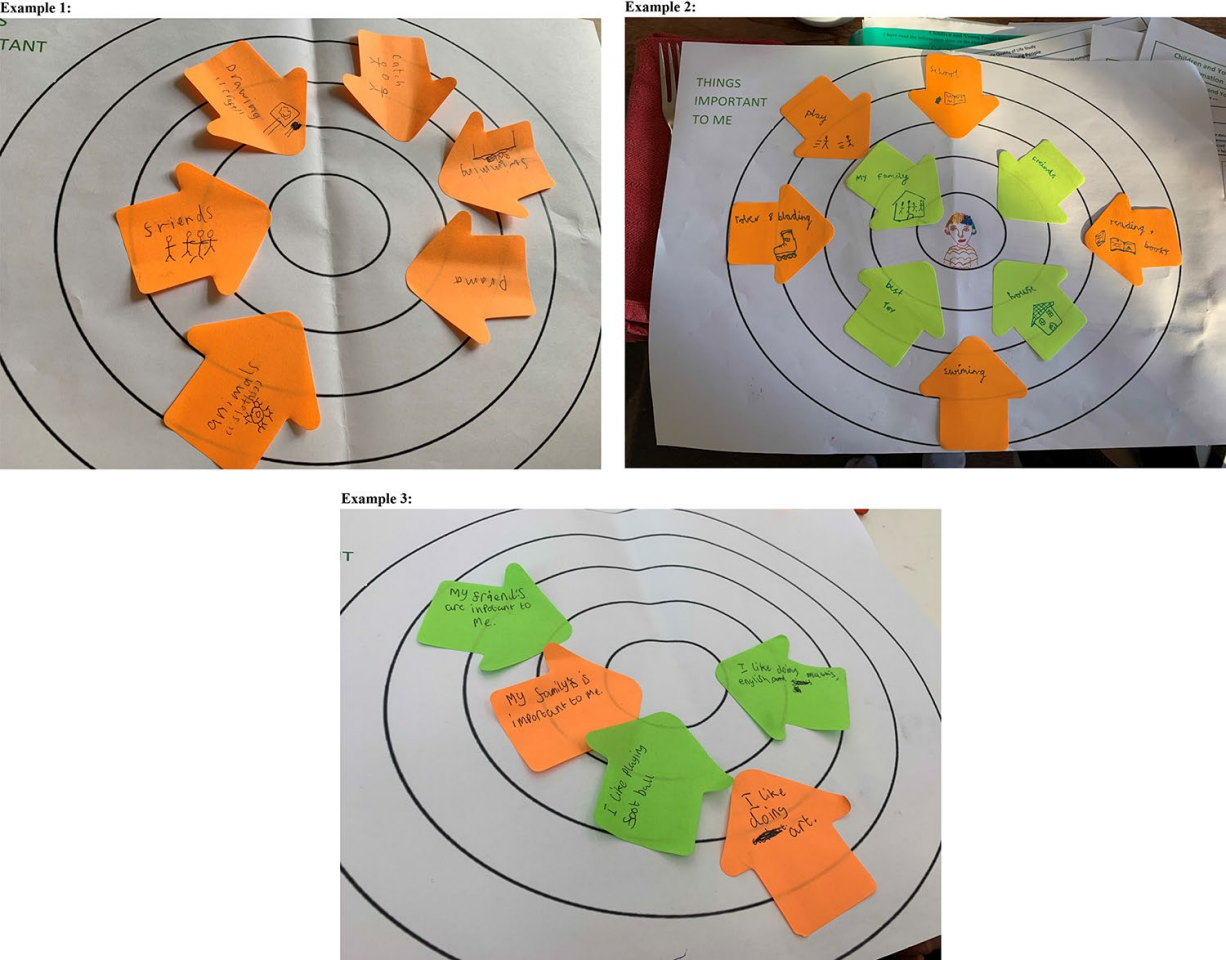
Examples of completed hierarchical mapping activities by children and young people informants

**Fig. 2:**
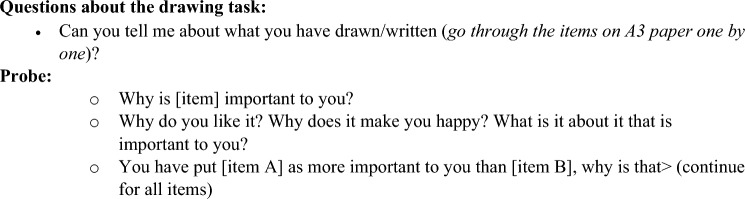
Questions from the interview topic guide about the hierarchical mapping activity

CYP were given an A3 sheet of paper featuring five concentric circles and asked to draw or take a picture of themselves and place this in the centre circle. During in-person interviews, CYP were invited to take a picture of themselves using a polaroid camera, providing an immediate image for use in the research. CYP were asked to draw or write things of importance to them on sticky labels and place these onto the A3 paper around the centre circle, placing the things of most importance closest to the centre and factors of less importance further away (in the outer circles) (see Fig. 1). The method was designed to elicit discussion around why CYP considered these factors important to their wellbeing, allowing these to be explored further during interviews (see Fig. 2 for an interview topic guide). The task was piloted with the YPAG to ensure that it was considered age appropriate for use with target CYP. Piloting the activity with the YPAG suggested that the activity was well received by CYP aged 10–17 years, as all members completed the task in the anticipated way without any difficulty. YPAG members suggested that giving CYP the freedom to write and/or draw what was important to them would be appealing to a broader range of CYP, including the youngest (aged 6 years) and eldest (aged 15 years) informants in our sample.

We wanted to ensure that using a CAP method would not distract from the aims of the research and would generate meaningful and interpretable data [23, 35]. Using CAP methods as the sole focus of data collection could lead to issues around interpreting data and whether researchers’ interpretations can be a true reflection of CYP’s views. We decided that the hierarchical mapping task should be used as a tool to stimulate discussion from CYP regarding what was important to them, to be followed by interview questions. CYP’s explanations in the interviews were key to understanding and developing the broader concepts underlying what the CYP considered to be important to generate the measure items.

Our experience of conducting over 40 interviews with CYP demonstrated that the activity was well-received across the broad range of CYP, including facilitating the involvement of relatively young children. Engagement was facilitated by a clear explanation of the activity prior to commencement, with the researcher ensuring that CYP understood that their answers could not be wrong. CYP were asked to think of all the things that were important to them, placing the things that were ‘most important’ to them closest to the picture of themselves in the middle and the ‘things that are important but not as important’ to them in the outer circles. All CYP, including the youngest in the sample, appeared able to understand the task and talk about the relative importance of the factors that they had recorded on their maps; however, it should be noted that other CYP in the younger age group, or even older, might struggle with this, and thus in these circumstances it might be more appropriate to simplify the activity and remove the ranking aspect.

CYP fed back to suggest that they enjoyed the activity and its creative element, particularly being able to take and keep their own photograph using the polaroid camera. On beginning the activity, many of the CYP, particularly those in the younger age groups, were visibly keen to use the camera, which helped to initially engage them in the study. The younger CYP also appeared to enjoy selecting colourful pens and notes to draw or write with. Interestingly, many of the younger CYP selected to write what was important to them as it appeared that they wanted to ‘show off’ their writing skills, which added another mechanism for engaging CYP in the activity. CYP at the older end of the age range tended to write their responses, although some did also draw what was important to them, suggesting that the nature of responses might be influenced by personal preferences and not only age. The older CYP all appeared happy to complete the activity, which alleviated concerns that older CYP might find being asked to complete a creative task patronising [23]. Having their completed maps to refer to during the interview seemed to increase CYP’s confidence when answering interview questions, as CYP regularly referred back to it when asked by the interviewer what they would next like to discuss.

Unfortunately, we did not officially collect or record feedback from the CYP on the activity, although we regularly observed how well it appeared to be engaging CYP and checked that they had enjoyed the task after the interview was complete. However, an alternative way to measure engagement is by observing and reporting the average length of data collection for CYP interviews, as this can provide an indication of how responsive CYP were and how rich emerging findings are likely to be [36–38]. In our study, the activity appeared to encourage prolonged and in-depth discussions from the CYP, as interviews following the hierarchical mapping activity lasted between 18 and 60 min (average 32.5 min). This is notably longer than the lengths of data collection reported for similar concept elicitation studies with CYP that did not use creative methods during data collection [8].

However, there are practical considerations to using CAP methods. Our activity was fairly low cost, with only the costs of the polaroid camera, pens, sticky labels, and printing required, but the activity added extra time to data collection, as CYP were given as long as they needed to complete the activity prior to the interview. We found that CYP typically took no longer than 10 min each to do the activity, but this could vary across all CYP and will add time on to the study duration overall. When data collection had to go online because of COVID-19, we found that we were unable to continue using the polaroid camera, which took away an engaging aspect of our study design. However, we posted out the hierarchical maps and sticky notes in advance of data collection to allow CYP to continue to complete the hierarchical mapping activity, and there appeared to be no difference in the richness of data collected after this change was made.

## Discussion

This paper has given detailed insight into key stages of qualitative concept elicitation for CYP. It has provided methodological detail on previously underreported steps [8, 11], particularly those that are unique to concept elicitation with CYP. Based on our own experiences of concept elicitation, we have offered insight into specific challenges with doing this research with CYP and the approaches that we have taken to navigate such challenges. In sampling and recruiting CYP, we have outlined the techniques that we used to navigate gatekeeping organisations; for example, using readily established links with organisations and taking recruitment online to sample CYP directly through their parents/guardians. We detail the steps that we took to ensure the appropriate and inclusive involvement of CYP in the concept elicitation study, including consulting a YPAG to develop suitable study documents. To encourage CYP to have freedom over their decision to take part in the study, we outline the processes that we followed to gain informed assent directly from each child or young person, with these processes not typically being covered in other empirical papers [8]. We have documented the use of a CAP activity that we adapted for concept elicitation with CYP, specifically the use of drawing, photographs, and hierarchical mapping techniques to generate concepts of importance to CYP to inform measure items. Our own reflections on the method suggest that several aspects of the activity were engaging for CYP, including use of the polaroid camera and being able to design their own hierarchical maps to share with the researcher. Informal feedback from CYP on the task and interview experience was positive and being able to refer to the completed maps in the interview appeared to encourage prolonged and rich data collection. We also aimed to use the method to minimise power differentials between the adult researcher and CYP, and we suggest that this approach worked well in giving the CYP control over the direction of the research through the use of their completed maps to guide what was discussed in the interview.

A strength of this paper is that, to the authors’ knowledge, it is unique in its in-depth and ‘real world’ coverage of key and challenging stages of qualitative concept elicitation with CYP that have not been addressed extensively elsewhere. Other studies have provided comprehensive overviews of the entire concept elicitation process [6, 7] or have focused on a specific issue, such as interviewing CYP for PROM development [24, 39], but particular topics such as whether CAP methods should be used to facilitate item generation have been left open for further exploration. We have also aimed to highlight the benefit of transparency in reporting concept elicitation with CYP, not only to facilitate judgements of content validity but also to allow research teams to learn from one another’s experiences. We anticipate that in providing such an in-depth overview of the methods that we used in our concept elicitation work we have generated a resource that is likely to be valuable to other researchers undertaking similar studies.

A limitation of our study is that it focuses only on our experiences of concept elicitation, which may mean that our reflections are less relevant in different measure development contexts. For example, if other research teams were to carry out concept elicitation with different groups of CYP, including with those in younger age groups, the CAP methods that we have presented might need to be adapted. We have also presented approaches to concept elicitation that do not necessarily complement one another; for example, in undertaking recruitment of CYP online to navigate organisational gatekeepers, key aspects of the CAP activity cannot be carried out, particularly use of the polaroid camera. If following similar methods, other researchers would therefore have to decide which approach would be most fruitful for them to take. Finally, we have presented methods that we have adapted for concept elicitation with CYP that have not been formally evaluated. This said, we have not aimed for this paper to be a prescriptive or comprehensive resource, and many high-quality guidelines already exist that researchers can refer to for developing and evaluating PROMs [6, 7, 40]. Instead, we have aimed to address the issue of low-quality reporting of concept elicitation with CYP by presenting our own methods in detail in the hope that other researchers will consider such transparency to be beneficial. Previous studies have reported a lack of detail and transparency in concept elicitation with CYP [8, 11], despite the availability of high-quality reporting guidelines for concept elicitation [41] and guidelines for reporting formative qualitative research in instrument development [42, 43]. Suggestions for enhancing the quality of reporting of concept elicitation work with CYP include the development of specific reporting guidelines, to raise awareness of the importance of transparent reporting for CYP measures [8]. However, many of the reporting principles outlined in general PROMs reporting guidelines are likely to also apply closely to reporting the development of instruments for CYP. Researchers may perceive that there is not the space available in journals to report in-depth methods, and thus publishing dedicated methods papers such as ours and the qualitative concept elicitation work by Stevens [44] might help to raise reporting standards generally. Depending on journal requirements, research teams could also make use of appendices to include more in-depth information on development stages, and attach a completed qualitative checklist with published papers such as the Consolidated Criteria for Reporting Qualitative Research (COREQ) [37], to demonstrate to potential measure users that key information has been detailed.

## Conclusion

This paper has provided detailed insight into unique and challenging stages of qualitative concept elicitation for PROM development with CYP. This is with a particular focus on previously underreported areas, including sampling and recruitment of CYP, encouraging informed assent and CYP’s freedom over their involvement in concept elicitation research, and the use of CAP methods to facilitate item generation. In developing this paper, we have aimed to provide a useful resource to other teams undertaking similar work while also reiterating the value of transparent and high-quality reporting in concept elicitation work with CYP.

## Declarations

### Data availability

Data sharing is not applicable to this article as no datasets were generated or analysed during the current study.

## References

[1] Drummond MF (2015). Methods for the economic evaluation of health care programmes.

[2] Kingsley C, Patel S (2017). Patient-reported outcome measures and patient-reported experience measures. BJA Educ..

[3] Weldring T, Smith SM (2013). Patient-reported outcomes (PROs) and patient-reported outcome measures (PROMs). Health Serv Insights..

[4] Patrick DL (2011). Content validity-establishing and reporting the evidence in newly developed patient-reported outcomes (PRO) instruments for medical product evaluation: ISPOR PRO Good Research Practices Task Force Report: part 1-eliciting concepts for a new PRO Instrument. Value Health..

[5] US FDA, Department of Health and Human Services. Patient-Reported Outcome Measures: Use in Medical Product Development to Support Labeling Claims: Guidance for Industry. Silver Spring: US FDA; 2009. 10.1186/1477-7525-4-79 PMC1629006 17034633

[6] Matza LS (2013). Pediatric patient-reported outcome instruments for research to support medical product labeling: report of the ISPOR PRO good research practices for the assessment of children and adolescents task force. Value Health..

[7] Arbuckle R, Abetz-Webb L (2013). "Not just little adults": qualitative methods to support the development of pediatric patient-reported outcomes. Patient.

[8] Husbands S, Mitchell PM, Coast J (2020). A systematic review of the use and quality of qualitative methods in concept elicitation for measures with children and young people. Patient.

[9] Coad J (2007). Using art-based techniques in engaging children and young people in health care consultations and/or research. J Res Nurs.

[10] Shaw C, Brady LM, Davey C. Guidelines for research with children and young people. London: National Children’s Bureau (NCB) Research Centre; 2011

[11] Willis J (2021). Engaging the voices of children: a scoping review of how children and adolescents are involved in the development of quality-of-life-related measures. Value Health..

[12] Brazier JE (2012). Developing and testing methods for deriving preference-based measures of health from condition-specific measures (and other patient-based measures of outcome). Health Technol Assess.

[13] Husbands S, et al. The children and young people quality of life study: a protocol for the qualitative development of attributes for capability wellbeing measures for use in health economic evaluation with children and young people. Wellcome Open Res. 2022;7:117.

[14] Mitchell PM (2021). Challenges in developing capability measures for children and young people for use in the economic evaluation of health and care interventions. Health Econ.

[15] Sen A, Nussbaum M, Sen A (1993). Capability and well-being. The quality of life.

[16] Patrick DL (2011). Content validity–establishing and reporting the evidence in newly developed patient-reported outcomes (PRO) instruments for medical product evaluation: ISPOR PRO Good Research Practices Task Force report: part 2—assessing respondent understanding. Value Health..

[17] Merkens H, Flick U, Kardorff EV, Steinke I (2004). Selection procedures, sampling, case construction. A companion to qualitative research.

[18] Carter SM (2021). Conducting qualitative research online: challenges and solutions. Patient.

[19] MacDonald A (2013). Researching with young children: considering issues of ethics and engagement. Contemp Issues Early Child.

[20] Guillemin M, Gillam L (2004). Ethics, reflexivity, and “ethically important moments” in research. Qual Inq.

[21] Phelan SK, Kinsella EA (2013). Picture this . . . safety, dignity, and voice—ethical research with children: practical considerations for the reflexive researcher. Qual Inq.

[22] Barker J, Weller S (2003). "Is it fun?" developing children centred research methods. Int J Sociol Soc Policy.

[23] Punch S (2002). Research with children: the same or different from research with adults?. Childhood.

[24] Patel ZS, Jensen SE, Lai J-S (2016). Considerations for conducting qualitative research with pediatric patients for the purpose of PRO development. Qual Life Res.

[25] Kirk S (2007). Methodological and ethical issues in conducting qualitative research with children and young people: a literature review. Int J Nurs Stud.

[26] Morrow V, Richards M (1996). The ethics of social research with children: an overview. Children Soc..

[27] Lasch KE (2010). PRO development: rigorous qualitative research as the crucial foundation. Qual Life Res.

[28] Whale K. The use of Skype and telephone interviews in sensitive qualitative research with young people: experiences from the ROCCA continence study. Qualitative Methods in Psychology Bulletin. 2017. p. 23.

[29] Angell R, Angell C (2013). More than just "snap, crackle, and pop": "draw, write, and tell": an innovative research method with young children. J Advert Res.

[30] Bradding A, Horstman M (1999). Using the write and draw technique with children. Eur J Oncol Nurs.

[31] Haijes HA, van Thiel GJMW (2016). Participatory methods in pediatric participatory research: a systematic review. Pediatr Res..

[32] Clark A, Statham J (2005). Listening to young children: experts in their own lives. Adopt Foster.

[33] Canaway A (2019). Close-person spill-overs in end-of-life care: using hierarchical mapping to identify whose outcomes to include in economic evaluations. Pharmacoeconomics.

[34] Antonucci TC (1986). Hierarchical mapping technique. Generations J Am Soc Aging..

[35] Fargas-Malet M (2010). Research with children: methodological issues and innovative techniques. J Early Child Res..

[36] Kitto SC, Chesters J, Grbich C (2008). Quality in qualitative research. Med J Aust.

[37] Tong A, Sainsbury P, Craig J. Consolidated criteria for reporting qualitative research (COREQ): a 32-item checklist for interviews and focus groups. Int J Qual Health Care. 2007;19(6):349–357. 10.1093/intqhc/mzm042 17872937

[38] Stenfors T, Kajamaa A, Bennett D. How to… assess the quality of qualitative research. Clin Teach. 2020;17(6):596–599. 10.1111/tct.13242 32790137

[39] Brédart A (2014). Interviewing to develop patient-reported outcome (PRO) measures for clinical research: eliciting patients’ experience. Health Qual Life Outcomes.

[40] Terwee CB (2018). COSMIN methodology for assessing the content validity of PROMs–user manual.

[41] Gagnier JJ (2021). COSMIN reporting guideline for studies on measurement properties of patient-reported outcome measures. Qual Life Res.

[42] Hollin IL (2020). Reporting formative qualitative research to support the development of quantitative preference study protocols and corresponding survey instruments: guidelines for authors and reviewers. Patient.

[43] Coast J (2012). Using qualitative methods for attribute development for discrete choice experiments: issues and recommendations. Health Econ.

[44] Stevens KJ (2010). Working with children to develop dimensions for a preference-based, generic, pediatric, health-related quality-of-life measure. Qual Health Res.

